# Income inequality and cardiovascular disease risk factors in a highly unequal country: a fixed-effects analysis from South Africa

**DOI:** 10.1186/s12939-018-0741-0

**Published:** 2018-03-06

**Authors:** Kafui Adjaye-Gbewonyo, Ichiro Kawachi, S. V. Subramanian, Mauricio Avendano

**Affiliations:** 1 Department of Social and Behavioral Sciences, Harvard T. H. Chan School of Public Health, 677 Huntington Avenue, Kresge 7th Floor, Boston, MA 02115 USA; 2 Department of Global Health and Social Medicine, King’s College London, Strand Campus, Strand, London, WC2R2LS UK

**Keywords:** Cardiovascular disease, Gini coefficient, Health behavior, Income inequality, Longitudinal, Multilevel, Risk factor

## Abstract

**Background:**

Chronic stress associated with high income inequality has been hypothesized to increase CVD risk and other adverse health outcomes. However, most evidence comes from high-income countries, and there is limited evidence on the link between income inequality and biomarkers of chronic stress and risk for CVD. This study examines how changes in income inequality over recent years relate to changes in CVD risk factors in South Africa, home to some of the highest levels of income inequality globally.

**Methods:**

We linked longitudinal data from 9356 individuals interviewed in the 2008 and 2012 National Income Dynamics Study to district-level Gini coefficients estimated from census and survey data. We investigated whether subnational district income inequality was associated with several modifiable risk factors for cardiovascular disease (CVD) in South Africa, including body mass index (BMI), waist circumference, blood pressure, physical inactivity, smoking, and high alcohol consumption. We ran individual fixed-effects models to examine the association between changes in income inequality and changes in CVD risk factors over time. Linear models were used for continuous metabolic outcomes while conditional Poisson models were used to estimate risk ratios for dichotomous behavioral outcomes.

**Results:**

Both income inequality and prevalence of most CVD risk factors increased over the period of study. In longitudinal fixed-effects models, changes in district Gini coefficients were not significantly associated with changes in CVD risk factors.

**Conclusions:**

Our findings do not support the hypothesis that subnational district income inequality is associated with CVD risk factors within the high-inequality setting of South Africa.

**Electronic supplementary material:**

The online version of this article (10.1186/s12939-018-0741-0) contains supplementary material, which is available to authorized users.

## Background

Income inequality has been hypothesized to affect risk for cardiovascular disease (CVD) through several pathways [1–4]. For example, physiological changes due to the chronic stress of increased social dysfunction in unequal communities can raise blood pressures or lead to the adoption of unhealthy coping behaviors (e.g., smoking, unhealthy eating, alcohol consumption), which can impact cardiovascular and other chronic diseases [5–13]. Additionally, income inequality has been consistently linked to heightened crime which has also been tied to reduced social cohesion [3, 14–16]. The perceived lack of safety resulting from high crime and low cohesion may reduce outdoor physical activity, leading to increased body mass index (BMI), blood pressure, and other cardiovascular risk [7].

However, the research testing the hypothesis that income inequality has a contextual effect on cardiovascular health and other adverse health outcomes—in other words that it affects health independently of individual or household income—has so far produced inconsistent results [12, 17–24], leaving the income inequality hypothesis at a bit of an impasse. Several explanations have been offered for the inconsistent findings. Wilkinson and Pickett [25, 26] observed that studies of the relationship between national income inequality and health are more consistently supportive of the income inequality hypothesis than those at sub-national levels [2, 17, 25]. They argue that income inequality is inherently a macro-level phenomenon and that this is because at increasingly smaller areas, income inequality and heterogeneity within an area is converted into differences in absolute income between areas, due to the greater homogeneity of residents in smaller and smaller units [25, 26]. Thus, health differences between small areas tend to reflect absolute income or deprivation differences between these areas rather than the inequality at the larger scale.

However, based on hypothesized mechanisms, it is still meaningful to test the influence of subnational income inequality on health and social outcomes. For example, links between inequality and crime have been established at local levels as well as on a larger scale [14, 27]. Moreover, the majority of studies of large subnational areas have still been supportive of the income inequality hypothesis [25, 26]. Fairly robust results from the U.S. as well as less consistent results from other settings [24, 28] suggest that local and subnational income inequality (at state, county, and municipal levels) may matter for health. Additionally, from a policy perspective, changes to income distributions are likely to be implemented within rather than across nations.

Research on subnational income inequality and health from within high-income countries has generally supported the relationship between income inequality and poor health in the U.S.—where Gini coefficients range between about 0.4–0.6. However, similar research within and across some European countries, in which Gini coefficients are generally lower than 0.4, has not supported the relationship. Some researchers have therefore suggested that this pattern may be indicative of a potentially non-linear effect of income inequality on health, whereby income inequality is detrimental to health only above a certain threshold [17, 22, 28–30]. This hypothesis therefore offers an alternative interpretation to that of Wilkinson and Pickett to explain the inconsistent support for the income inequality hypothesis in empirical studies.

However, there is limited research among highly unequal localities (Gini coefficients above 0.6) to elucidate these competing hypotheses and examine the potential form of the income inequality and health relationship at the upper end of the income inequality spectrum. Many high inequality countries are low- and middle-income countries (LMICs) [4], and so far, associations between inequality and poor health in LMICs such as Brazil, Mexico, and South Africa have been inconsistent [31–34]. Complicating matters, the question remains as to whether income inequality and relative income matter for health mostly in high-income settings, where basic needs have largely been met (as has been implied by much of the work from Wilkinson and Pickett), or whether it still matters for health in settings in which income is low and poverty is high, such as in several LMICs [35].

### Income inequality and cardiovascular risk in South Africa

The middle-income country of South Africa, presents an interesting case for the study of inequality. Arguably due to the legacy of colonialism and apartheid [36, 37], South Africa is one of the most unequal societies in the world. Based on the most recent World Bank data, South Africa’s 2011 Gini coefficient of 0.65 was the highest among countries with available data [38]. Moreover, there is evidence that income inequality has increased post-apartheid [36, 37, 39]. High income inequality in South Africa has been offered as an explanation for the country’s comparatively low life expectancy, high crime and homicide rates, low social mobility, high levels of self-enhancement, and low levels of trust [14, 15, 40–42].

Additionally, in spite of residential segregation, largely on racial and consequently economic lines, income inequality remains extremely high in South Africa even at subnational levels. As our analysis of census data below demonstrates, at district levels (2011 district average Gini coefficient: 0.75, range: 0.69–0.78), inequality is nearly on par with that observed on a national level (2011 national Gini coefficient: 0.78). South Africa, a high-inequality, middle-income country, thus offers a unique setting to study income inequality and its relation to health and elucidate some of the remaining questions about the scales at which income inequality operates on health (e.g., subnational as compared to national) and the levels or ranges in which changes in income inequality matter for health (e.g., at low, middle, or high levels of inequality).

If findings regarding income inequality and health at subnational levels in a very high inequality country such as South Africa support the income inequality hypothesis, it may provide additional clues as to the contexts in which changes in income inequality matter for health and help to interpret the heretofore mixed support for the income inequality hypothesis in the empirical evidence. This would indicate that it is not necessarily the case that income inequality does not operate on health at smaller areas, but rather that it is characteristics of these areas, such as levels of inequality, that may determine whether changes in income inequality may have an effect on health.

To address these questions, we use data spanning a four-year time span between Waves 1 and 3 of the National Income Dynamics Study (NIDS), therefore exploring the potential effects of changes in income inequality of a magnitude that may be reasonably achievable through policy in low- and middle-income settings. We examine these potential effects on short- to medium-term health outcomes, specifically risk factors for cardiovascular disease. Unlike health outcomes such as mortality or disease which may in some cases take years to manifest after exposure to inequality [12, 43, 44], risk factors such as increased blood pressure, body weight, or unhealthy behaviors may be expected to change over shorter periods of time.

Risk for CVD is an important health concern in South Africa. There are several indications that the burden of non-communicable diseases in South Africa is rising. In 2010, circulatory or cardiovascular causes were the second leading cause of death in South Africa (after infectious and parasitic illnesses), and, the proportion of deaths due to these causes has increased over time [45]. CVD risk factors such as overweight and obesity are common in South Africa, with around 60% of women and 30% of men being classified as overweight, and approximately one-third of women and 10% of men being classified as obese [46, 47]. Excessive alcohol consumption and high blood pressure are also highly prevalent [48–50]. However, unlike in high-income countries such as the U.S., risk factors such as overweight and obesity, while widespread, have tended to follow patterns observed in other LMICs, being more prevalent among individuals with higher incomes and education levels as compared to individuals with lower socioeconomic status [46, 48, 50, 51].

### Study objectives

In the present study, we attempt to address some of the aforementioned gaps in the literature by using longitudinal data from South Africa to examine whether changes in district-level income inequality are associated with changes in individual-level risk factors for CVD. We use fixed-effects models that exploit variations in income inequality between 2007 and 2011 to control for the stable effects of time-invariant confounders and enhance causal inference. To our knowledge, this is the first longitudinal study to examine the question of the association between changes in income inequality and CVD risk factors in the African continent. We hypothesize that in the South African context, increases in district income inequality will be associated with worsening indicators of CVD risk. Moreover, we expect the relationship between income inequality and health to be as strong or stronger in a very unequal country such as South Africa compared to the United States or to European countries.

## Methods

### Data sources

#### National income dynamics study

Data were drawn from the NIDS, a nationally-representative, household, longitudinal survey conducted by the Southern Africa Labour and Development Research Unit (SALDRU) [52]. Sampling was done in a stratified, two-stage cluster sample design as described elsewhere, and the household response rate was 69% [53–56].

**Fig. 1:**
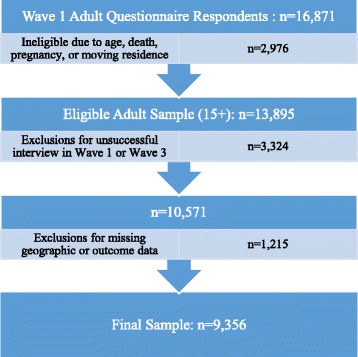
Flowchart of sample selection

There were 16,871 individuals aged 15 years or older from 7305 households who participated in the Wave 1 (2008) adult questionnaire. We excluded 2976 respondents who: a) had incomplete or discrepant age information or were under age 15 years at the time of interview in 2008 (*n* = 110); b) had died by Wave 3 (*n* = 1208); c) were living outside of South Africa in Wave 3 (*n* = 43); d) were living in a different district from their original Wave 1 district in Wave 3 (*n* = 1203); or e) were pregnant or had missing or unknown pregnancy status in Wave 1 (*n* = 359) or Wave 3 (*n* = 177). This resulted in an eligible sample of 13,895 respondents. Of these, 3324 respondents were excluded due to unsuccessful interviews or loss to follow-up [Wave 1 (*n* = 1045) and Wave 3 (*n* = 2564)], and an additional 1215 respondents were excluded due to either missing or unknown district information in Wave 3 (*n* = 27) or lack of data on any of the outcomes examined in either Wave 1 or Wave 3 (*n* = 1215) after exclusions for extreme values. Our final sample contained 9356 individuals (see flowchart in Fig. 1).

#### District-level data

South Africa is divided administratively into nine provinces, and further into 52 district councils/district municipalities and over 200 local municipalities, as of the Census 2011 [56–58]. Our study focuses on income inequality at the district level.

**Fig. 2:**
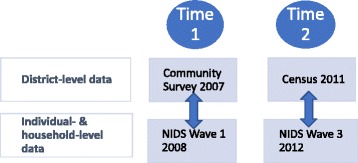
Diagram of the merging of census and survey data with the NIDS

Data for district variables were calculated from South Africa’s Community Survey 2007 (CS 2007) [59], and from a 10% sample of the Census 2011 [60]. The CS 2007 consists of a 2% random population sample surveyed by Statistics South Africa (Stats SA) to provide information between the 2001 and 2011 censuses and to provide data for municipalities and other subnational regions. The CS 2007 contains data from 238,067 dwelling units or approximately one million persons and had a response rate of 93.9% [59, 61]. District-level data calculated from the CS 2007 were matched to individual-level data from the 2008 NIDS Wave 1 survey for the present analysis (Fig. 2).

The 10% sample of the Census 2011 contains over one million households and nearly 4.5 million individuals [58]. Data from the census sample were matched to the 2012 NIDS Wave 3 survey (Fig. 2). District boundaries changed in 2011 [57, 62], so for consistency, districts were constructed from the CS 2007 to correspond with the 52 districts defined in the Census 2011. A list of districts is displayed in Additional file 1.

### Income inequality

We measured income inequality using the Gini coefficient [20]. Gini coefficients were calculated in SAS [63] from gross income (before deductions but including social grants) as reported in the Census 2011 and CS 2007 [64]. Incomes were deflated to August 2012 prices and equivalized by dividing by the square root of household size. Details of these calculations are provided in Additional file 2. We multiplied Gini coefficients by a factor of 10 for use in the models so that the interpretation of the model estimates would correspond to each change in Gini coefficient of 0.10 points.

### Cardiovascular risk factors

Our study focused on the following major modifiable risk factors for CVD [65, 66]:

### Metabolic risk factors

#### Blood pressure

Blood pressure was measured in the NIDS using two readings. We calculated systolic blood pressure (SBP) and diastolic blood pressure (DBP) as the means of the plausible readings in each wave.^1^

#### BMI

Height and weight were measured in each NIDS survey using three readings each. We calculated BMIs as mean weight in kilograms divided by the square of mean height in meters. Mean weights less than or equal to 31.8 kg (approximately 70 pounds) or greater than or equal to 150 kg (approximately 331 lbs.) and mean heights less than or equal to 121.9 cm (48 in.) or greater than 213.4 cm (84 in.) were considered implausible and excluded [67, 68]. Mean heights that were implausible, missing, or differed by more than 10 cm between any two waves were replaced with the mean of the plausible average heights across the three waves where available.

#### Waist circumference

Waist circumference in centimeters was measured using up to three readings in each wave. We used the mean of the plausible waist circumference measurements in each wave for our analysis.^2^

### Behavioral risk factors

#### Smoking

Smoking was self-reported. To facilitate analysis of smoking as a categorical outcome using risk ratios, and because of the small number of former smokers, we dichotomized smoking into two categories--current smokers and current non-smokers.

#### Alcohol consumption

We estimated the average number of drinks consumed per day using the frequency of alcohol consumption per week and the number of standard drinks consumed per day of drinking [69] (see Additional file 2). High alcohol consumers were defined as respondents who reported drinking five or more standard drinks on a day when they drink alcohol, women who averaged more than one drink per day or eight or more drinks per week, and men who averaged more than two drinks per day or 15 or more drinks per week [70].

#### Physical inactivity

Respondents were asked how many days a week they exercised. We defined physical inactivity as no days a week (versus one or more days a week) of exercise.

### Covariates

Individual- and household-level covariates from the NIDS included the following time-varying variables: employment status (employed, unemployed, not economically active); marital status (currently married/cohabiting or currently single), receipt of at least one government grant by a household member (old age pension, child support grant, or other social grants) [71], and household income based on post-tax household income in Rand over the past 30 days [71]. We deflated incomes to August 2012 levels [72, 73]. To account for the non-linear relationship between income and health, we used the natural log of income in our models.^3^ Time-constant variables (sex, race/population group), variables collinear with time (age), as well as variables with little change over time (rural/urban location and education) were excluded from fixed-effects models.

To control for potential confounding effects of district characteristics, we calculated the following district covariates from the CS 2007 and Census 2011 using individual or household weights as applicable: log of mean equivalized monthly household income; percent of persons aged 15+ years with no education and with higher education; percent of persons aged 15–65 years who are unemployed; percent of households that are rural; mean age; percent African; and percent female. There was evidence of partial collinearity between mean district household income and other variables (variance inflation). However, because it is important to distinguish between the effects of district income levels and district income inequality, we retained the log of mean district household income in the models.

### Analysis

For all variables, refusals and missing, “don’t know” or “not applicable” responses were coded as missing. For each model, individuals with missing covariates (< 3% of sample) were excluded.

For data in a multilevel structure, such as that used in the present analysis (e.g. individuals within districts), random or mixed-effects models—also known as multilevel or hierarchical models—are a commonly-used method to account for the non-independence or clustering of observations and to estimate the contributions of each level of data to the variation in the outcomes. This form of regression would examine the between-individual and between-district variation in outcomes. However, such models may be susceptible to bias and unobserved confounding arising from these between-unit differences. To address this, we therefore implemented longitudinal fixed-effects models on individuals present in both Waves 1 and 3. Fixed-effects models exploit the longitudinal nature of the data by relating within-individual changes in outcomes to within-individual changes in exposure. They are an attractive method for attempting to estimate causal effects because they account for any unobserved confounders that are constant and have stable effects over time. Fixed-effects models use each individual as his or her own control, by comparing an individual’s health when exposed to a given level of district income inequality, with the same individual’s health when exposed to a different level of district income inequality. Assuming that intra-individual changes in income inequality are uncorrelated with changes in other variables, within-individual changes in health over time provide an estimate of the effect of income inequality on health outcomes. Fixed-effects estimates obtain the average differences across all individuals to yield an estimate of the average ‘treatment effect’ of district income inequality, which in this case controls for all stable individual and district characteristics. Because fixed-effects models do not control for characteristics that change over time, our models still incorporate a wide range of time-varying district- and individual-level measured covariates as described above.

The models were specified as follows:

1 $$ {\displaystyle \begin{array}{c} CVD\ {Risk}_{tihj}={\beta}_0+{\beta}_1{Inequality}_{tj}+\kern0.5em {\beta}_2{Covariates}_{tihj}+{\beta}_3{Covariates}_{thj}\\ {}+{\beta}_4{Covariates}_{tj}+{\beta}_5{wave}_t+{\beta}_6{individual}_{ihj}+{e}_{0\mathrm{t} ihj}\end{array}} $$

where *t* represents wave, *i* represents individuals, *h* represents households, and *j* represents districts. *CVD Risk*_*tihj*_ represents the individual-level CVD risk factors in Waves 1 and 3. *β*_0_ is the intercept. *Inequality*_*tj*_ is district income inequality in each wave. *Covariates*_*tihj*_, *Covariates*_*thj*_, and *Covariates*_*tj*_ are vectors of individual-, household-, and district-level covariates, respectively. *Wave*_*t*_ indicates time fixed-effects. *Individual*_*ihj*_ represents individual fixed-effects, and *e*_0t*ihj*_ are the error terms. All variables that are constant over time—including unmeasured confounders—drop out of the models. The main effect of interest was measured by coefficient *β*_1_.

For continuous metabolic outcomes (BMI, waist circumference, DBP, and SBP), we ran linear models with standard errors clustered by district. For dichotomous behavioral outcomes (high alcohol consumption, smoking, and physical inactivity), we ran Poisson regression models, rather than logistic regression, to directly estimate risk or prevalence ratios. Odds ratios from logistic regression only approximate risk ratios when outcomes are rare. In this sample, the behavioral outcomes were fairly common, so odds ratios would overestimate risk ratios. Regression methods such as log-binomial models and robust Poisson regression can therefore be used to estimate risk ratios. We selected robust Poisson regression for our dichotomous outcomes because, while Poisson regression may be more conservative than log-binomial models and other methods of estimating risk ratios, it tends not to have as much difficulty converging [74, 75]. To estimate the standard errors in the conditional (fixed-effects) Poisson regression models, we bootstrapped the standard errors using 200 replications, with clustering at the district level; we used normal-based confidence intervals [76–78].

For each outcome, models adjusting only for wave (Model 1), adding individual/household covariates (Model 2), and adding district covariates (Model 3) are presented. Sensitivity analyses using three-level random-intercept mixed-effects models (observations clustered within individuals clustered within districts) were also run.

Analyses were conducted in Stata versions 13 and 14 and SAS version 9.4.

## Results

### Descriptive statistics

**Fig. 3:**
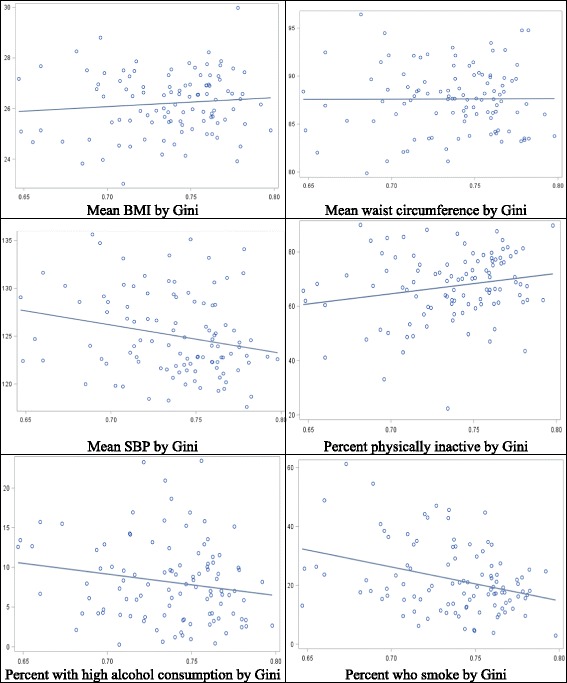
District-level prevalence of CVD risk factors (y-axis) by district Gini Coefficent (x axis), pooled Waves 1 and 3

The socio-demographic characteristics and health status of our sample are summarized in Table 1. The sample was predominantly female (63.0%), African (80.3%), and currently single (60.6%) with a mean age of 39 years at baseline. Over 18% of the sample were unemployed at baseline, and over 42% were not economically active; about 61% of households received government grants. More than half of households were rural. At baseline, the mean BMI was 26.3 kg/m^2^ (SD = 7.1 kg/m^2^), and mean waist circumference was 87.4 cm (SD = 16.2 cm). Mean SBP was 127.4 mmHg (SD = 23.7 mmHg), and mean DBP was 81.9 mmHg (SD = 14.3 mmHg) at baseline. Over 19% of the sample smoked at baseline, and more than 10% had high alcohol consumption. Nearly three-quarters of the sample reported that they did not exercise. Average outcome levels varied considerably by district (see Fig. 3).

**Table 1:** Sample characteristics, NIDS waves 1 and 3

	Wave 1		Wave 3	
	N	Proportion/Mean (Standard deviation)	N	Proportion/Mean (Standard deviation)
Total	9356		9356	
Female	5898	63.0%	5898	63.0%
Race/population group				
* African*	7514	80.3%	7514	80.3%
* Coloured*	1385	14.8%	1385	14.8%
* Asian/Indian*	110	1.2%	110	1.2%
* White*	347	3.7%	347	3.7%
Age (years)	9356	39.3 (17.3)	9356	43.5 (17.4)
Highest education level				
* No education*	1346	14.4%	1300	13.9%
* Some general education & training*	3355	35.9%	2990	32.0%
* General education & training*	828	8.9%	662	7.1%
* Some further education & training*	1929	20.6%	2120	22.7%
* Further education & training*	1309	14.0%	1303	13.9%
* Higher education*	582	6.2%	973	10.4%
Employment status				
* Employed*	3653	39.4%	3560	38.1%
* Unemployed*	1707	18.4%	1584	17.0%
* Not economically active*	3902	42.1%	4190	44.9%
Marital status				
* Currently Married/Cohabiting*	3572	39.4%	3628	38.8%
* Currently single*	5643	60.6%	5722	61.2%
Household size^a^	5318	4.3 (2.7)	5870	4.4 (2.9)
Household receipt of government grants^a^	3214	60.7%	3672	62.6%
Monthly household income (Rand)^a^	5318	5082.0 (8717.1)	5870	5884.4 (9720.5)
Rural household^a^	2724	51.2%	2992	51.0%
Systolic blood pressure (mm Hg)	8523	127.4 (23.7)	9172	127.9 (23.0)
Diastolic blood pressure (mm Hg)	8520	81.9 (14.3)	9172	83.5 (13.7)
Body mass index (kg/m^2^)	8507	26.3 (7.1)	9161	27.4 (6.7)
Waist circumference (cm)	8517	87.4 (16.2)	9140	91.1 (16.0)
Current smoker	1819	19.5%	1783	19.1%
Physical inactivity	6669	71.7%	6957	74.4%
High alcohol consumption	994	10.7%	1142	12.3%

^a^Sample size is number of households

**Fig. 4:**
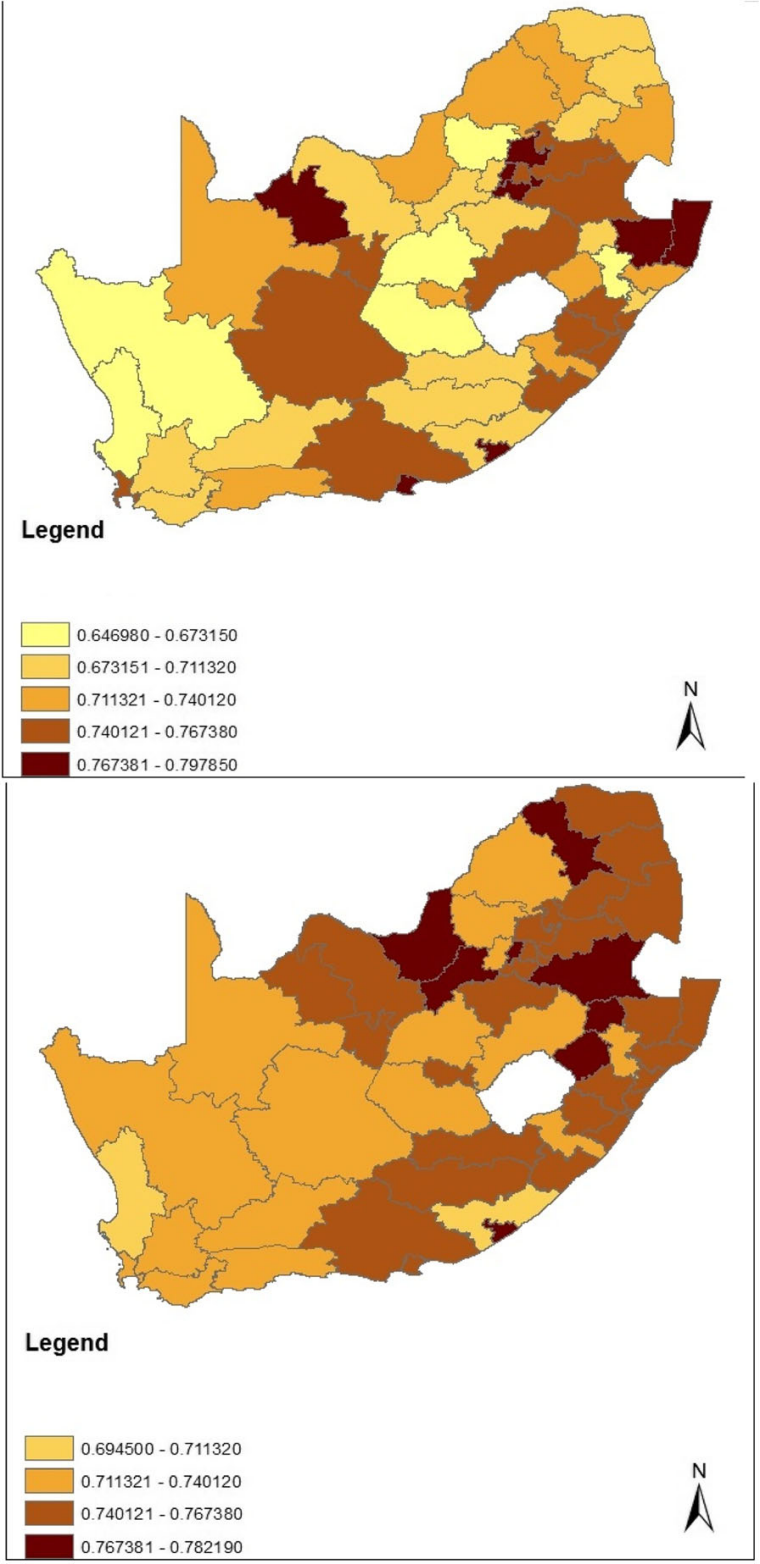
South Africa District Council Gini Coefficients, Community Survey 2007 (top) and Census 2011 (bottom)

In terms of income inequality, the average district Gini coefficient was 0.73 in 2007 (range: 0.65 to 0.80) and 0.75 in 2011 (range: 0.69 to 0.78). Figure 4 maps the Gini coefficients by district in 2007 and 2011. On average, district Gini coefficients increased by 0.02 points; however the change in district Gini across waves ranged from − 0.05 points for Umkhanyakude to 0.08 points for Xhariep. Detailed Gini coefficient information can be found in Additional file 1.

### Association between income inequality and CVD risk factors

Crude correlational analyses show that higher Gini coefficients were marginally correlated with higher mean BMIs and percentages of physically inactivity and with lower mean blood pressures and percentages of smoking and high-alcohol consumption at a district level (Fig. 3). However, regression results for CVD risk factors show that most of these associations did not hold in fully-adjusted models. In fixed-effects models controlling for stable effects of time-constant confounders, changes in district Gini were not significantly associated with changes in any of the CVD risk factors examined (Tables 2 and 3). Additional file 3 shows the estimates for other covariates in the models. Changes in individuals’ household income and in mean district income were not significantly associated with changes in CVD risk factors in fixed-effects models, although mixed-effects multilevel models showed that higher household incomes and individual education levels were associated with larger BMIs and waist circumferences and with lower risk of smoking and physical inactivity (Additional file 3).

**Table 2:** Effect estimates and 95% confidence intervals for the association between income inequality and metabolic CVD risk factors

	Longitudinal Fixed-Effects Models		
	Model 1^a^	Model 2^b^	Model 3^c^
BMI (kg/m^2^)	0.03 (−0.56, 0.64)	0.03 (− 0.56, 0.62)	0.26 (− 0.52, 1.03)
Waist circumference (cm)	−1.49 (−3.26, 0.27)	− 1.62 (− 3.33, 0.07)	−0.86 (− 3.39, 1.67)
Systolic blood pressure (mm Hg)	−1.66 (− 4.07, 0.76)	−1.57 (− 3.95, 0.82)	−1.14 (− 4.19, 1.90)
Diastolic blood pressure (mm Hg)	0.32 (− 1.47, 2.11)	0.30 (− 1.43, 2.03)	1.31 (− 1.17, 3.79)

Estimates correspond to a change of 0.10 in the Gini coefficient from linear fixed-effects models. Standard errors are clustered by district

^a^Controls for survey wave

^b^Controls for: wave; marital status, employment status; and household log household income, size, and receipt of government grants

^c^Adds district-level variables to Model 2: mean age; log mean monthly equivalized household income; and percents female, African, unemployed, with no education, with tertiary education, and rural

**Table 3:** Risk ratios and 95% confidence intervals for the association between income inequality and behavioral CVD risk factors

	Longitudinal Fixed-Effects Models		
	Model 1^a^	Model 2^b^	Model 3^c^
Smoking (risk ratio)	0.99 (0.84, 1.16)	0.97 (0.83, 1.15)	0.94 (0.74, 1.19)
High alcohol (risk ratio)	1.36 (0.93, 1.97)	1.35 (0.92, 1.97)	1.22 (0.54, 2.80)
Physical inactivity (risk ratio)	0.91 (0.82, 1.02)	0.92 (0.82, 1.01)	0.93 (0.77, 1.13)

Estimates correspond to a change of 0.10 in the Gini coefficient from conditional (fixed-effects) Poisson regression models. Standard errors are clustered by district

^a^Controls for survey wave

^b^Controls for: wave; marital status, employment status; and household log household income, size, and receipt of government grants

^c^Adds district-level variables to Model 2: mean age; log mean monthly equivalized household income; and percents female, African, unemployed, with no education, with tertiary education, and rural

## Discussion

South Africa is one of the most unequal nations in the world. Yet, we found that district-level income inequality, as measured by the Gini coefficient, was not significantly associated with CVD risk factors in this sample. While both Gini coefficients and several CVD risk factors increased slightly over time, the increases in CVD risk factors were not explained by changes in inequality. Our findings are in line with recent studies using fixed-effects methods that have failed to find associations between changes in inequality and changes in population health outcomes [22, 79, 80].

Thus, the results leave several questions unanswered in the study of income inequality and health. As has been suggested, subnational units, such as district councils, may not be the relevant units for examining the effects of income inequality on health. Alternatively, these results still support the argument of a possible non-linear relationship between income inequality and health. Income inequality was high for all districts in South Africa in both waves, and though Gini coefficients increased in many districts, it is possible that at the high levels of inequality observed in South Africa, there are no marginal effects of additional changes in inequality on these outcomes. This might imply that effects of income inequality have a ceiling above which additional effects are no longer observed. As previously discussed, it has already been suggested that there may be *floor* effects for the association between income inequality and health because some studies have not observed associations in low inequality settings but have in higher inequality countries such as the U.S. [22]. Thus, if both are true, then changes in income inequality would only affect health at medium levels of inequality.

An alternative interpretation is that the range of inequality is too narrow to observe effects, meaning that larger changes in inequality are needed to meaningfully impact health. For example, when Ross and colleagues [30] observed an association between income inequality and mortality across U.S. municipalities but not Canadian ones, not only were levels of inequality in Canada lower on average than in the U.S., the range was also smaller. Likewise, in our study, not only are inequality levels high, the range is also relatively modest. Thus, the apparent effect modification across study settings may not just reflect nonlinear effects based on the *level* of inequality, but may also be a reflection of the *range* and magnitude of changes in inequality needed to impact health. Therefore, even in a setting with income inequality levels like those in the U.S., it is possible that effects may not be observed across a narrower range or with small changes in inequality.

Still, other factors may have contributed to the findings. On the methodological front, our fixed-effects models may underestimate the impact of income inequality because they only exploit within-district variation in the Gini, while eliminating between-district variation, the largest share of total variation [43]. Effects of district inequality on CVD risk factors may be mediated by other variables in the models or by fixed individual, household, or district characteristics that are automatically controlled for in fixed-effects designs (e.g., individual educational attainment) [25]. However, even in our reduced fixed-effects models, no significant effects of changes in district inequality were observed. In addition, in sensitivity analyses using mixed-effects models with robust standard errors, results still did not support the hypothesis.

Alternatively, we may not have captured the relevant etiologic period. In the present analysis, Gini coefficients for each wave were based on data from the previous year which further referred to income during the preceding 12 months, thus, measuring effects over 1 to 2 years. We believe that the outcomes examined here—changes in blood pressure, weight gain or loss, and changes in alcohol consumption, smoking, and physical activity—may be responses to stress resulting from income inequality that manifest in a shorter time than outcomes such as mortality which have been shown to have longer-term associations with income inequality [44]. However, we may still have benefitted from more follow-up time. Nevertheless, (apart from Wave 3 DBP and physical inactivity), sensitivity analyses using cross-sectional models likewise failed to show detrimental effects of income inequality on CVD risk factors (not shown). Given that Gini coefficients often correlate highly over time, results from cross-sectional models may be attenuated but should generally reflect the long-term effects of inequality on health [28, 43]. Moreover, in sensitivity analyses matching the NIDS Wave 1 to Gini Coefficients from South Africa’s Census 2001 (7–8 years prior) and Wave 3 to Gini coefficients from the CS 2007 (5–6 years prior), only SBP and physical inactivity suggested potential adverse associations with district income inequality, but controlling for changes in district covariates eliminated these associations. Thus, even when using Gini coefficients from 5 to 8 years prior, there was not strong support for an adverse effect of district income inequality on these health outcomes, and given the number of analyses conducted, the few positive findings should be interpreted with caution.

Another consideration is that our Gini coefficients were based on pre-tax income available in the Census 2011 and CS 2007. It is possible that post-tax income may be more relevant for health outcomes. However, sensitivity analyses using Gini coefficients calculated from net, post-tax income in the NIDS still resulted in either no or inverse associations between inequality and CVD risk factors. Finally, another consideration is that Gini coefficients as a measure may not capture inequality effectively, as has been suggested in recent research [81].

A potential causal explanation is that there is no causal effect of income inequality on these specific CVD risk factors in South Africa. In the present sample, as in several LMICs (Subramanian et al. 2011), conditions such as high BMI or waist circumference are still predominantly associated with higher socioeconomic status (SES). Pickett and Wilkinson [25] argue that income inequality may amplify the prevalence of outcomes associated with *low* SES or with a strong inverse social gradient. Thus, it is possible that conditions related to poverty such as infectious diseases (e.g., HIV/AIDS and tuberculosis) and undernourishment may be more sensitive to inequality in South Africa than the outcomes examined here. Previous cross-sectional research has observed associations between Gini coefficients and tuberculosis in South Africa [34]. Likewise, some of our cross-sectional sensitivity analyses seemed to suggest inverse relationships between inequality and measures of bodyweight. Furthermore, Averett, Stacey and Wang [51] observed that province of residence explained some of the racial differences in underweight prevalence in South Africa but not in obesity prevalence, which could suggest that contextual factors might play a bigger role in undernutrition than overnutrition in this setting. Additional research is needed to examine this issue.

Finally, and more crucially, our results may indicate that effects of income inequality on health are confounded by other factors. Given that even interactions we explored between income and income inequality in this sample were not significant, the investigation of possible confounding in the income inequality and health relationship deserves further exploration.

### Strengths and limitations

Strengths of this study include a large sample size as well as the use of anthropometric measurements, including directly measured height, weight, and blood pressure, reducing the potential for self-report bias. Also, because the study uses within-country data, it may be less subject to confounding by the types of political, cultural, legal, and economic factors that may confound cross-country studies of inequality. In addition, using data from the CS 2007 and Census 2011 to calculate Gini coefficients and district covariates provided estimates that were representative at the district level. Income inequality was also measured before our health outcomes at both time points, thus establishing temporal order between exposure and outcome. Moreover, the socio-demographic, health, and income inequality distributions observed in our study are similar to those observed in other South African studies [36, 46, 48, 50, 51]. Finally, we were able to exploit the longitudinal nature of the NIDS to conduct fixed-effects analyses examining the associations between changes in district income inequality and changes in individual-level CVD risk factors over time, thus explicitly testing the hypothesis of contextual effects of income inequality on health within an unequal country while controlling for both observed and stable unobserved factors. Given that few studies of income inequality and health have applied the fixed-effects design while controlling for individual-level factors or have examined CVD-related outcomes in highly unequal countries in sub-Saharan Africa, this study extends previous research and offers a comparison with results observed in more equal, high-income nations.

Nevertheless, several limitations of this study should be considered. Although the NIDS was nationally-representative, the individuals excluded from this analysis were not missing completely at random. For example, at baseline, individuals excluded from the sample tended to differ from those included in terms of characteristics such as age, education, sex, race, residence, household size, incomes, and health outcomes (see Additional file 4 for baseline characteristics of those excluded from the analysis). This could affect not only the generalizability of our results but could introduce selection bias (or endogenous sampling) if inclusion in our sample varied in relation to the outcomes, conditional on the explanatory variables. However, we ran sensitivity analyses applying panel weights that adjusted for the probability of attrition based on gender, age, race/population group, province, marital status, and education [53]. These did not change the overall conclusions of this study.

District boundaries also changed slightly in 2011 [57, 62]. Though 2011 boundaries were used throughout this analysis, because data below the municipality level was not available in the CS 2007, and district management areas within each district were combined, this limited re-allocation of district management areas and within-municipality boundary changes in the CS 2007 for seven districts [57]. Models run excluding these districts yielded similar results, however, and the inclusion of district-level covariates in our models may have also helped to account for some of the differences due to boundary changes.

South African census data sources, including the CS 2007, have substantial percentages of households reporting zero income; therefore inequality may be overestimated in census sources [82]. Also, the CS 2007 did not include some institutionalized populations whereas the Census 2011 did [60, 64]. Nevertheless, the district estimates from the two data sources were very similar and highly correlated.

There was some evidence of inconsistencies or errors in the NIDS data, particularly for some of the anthropometric data in Wave 1 [46], as well as for education. Implausible values were excluded for anthropometric outcomes, and education was excluded in fixed-effects models to address this. Still, there may be data errors, which if systematic could bias the results, and if random could attenuate the results toward the null [83].

It should be noted that survey participants with high blood pressure readings were given information advising them about seeking treatment. This could attenuate associations observed in this analysis if treatment is sought between Waves 1 and 3. While variables on healthcare utilization were excluded from the analysis because of inconsistencies over time for several participants, sensitivity analyses controlling for antihypertensive use produced similar results for SBP and DBP outcomes to those presented here.

A final limitation is that the risk of residual time-varying confounding still remains, as in all observational studies, and even variables that are constant over time may have residual confounding if their effects on the exposure or outcome vary over time.

## Conclusion

Overall, our fixed-effects results did not provide support for an effect of changes in subnational district income inequality on CVD risk factors in South Africa, one of the most unequal countries in the world. Additional research may examine whether other outcomes, such as communicable diseases and poverty-related conditions, are linked to inequality in South Africa. Likewise, analyses over more extended periods, considering longer time lags, and encompassing broader ranges of inequality and a variety of units of analysis may yield different results and also elucidate issues such as potential ceiling and floor effects and effect modification by context. Such research may clarify some of the remaining debates in income inequality research regarding the geographic scales at which income inequality affects health and the ranges in which changes in inequality affect health, among other unanswered questions.

## Additional files

Additional file 1: National and district council Gini coefficients. (DOCX 15 kb)

Additional file 2: Additional details on variables used in analysis. (DOCX 22 kb)

Additional file 3: Model results. (XLSX 89 kb)

Additional file 4: Baseline characteristics for individuals excluded from the sample. (DOCX 20 kb)

## Abbreviations

BMI: Body mass index
CDC: Centers for Disease Control and Prevention
CS 2007: Community Survey 2007
CVD: Cardiovascular disease
LMIC: Low- or Middle-Income Country
NIDS: National Income Dynamics Study
SALDRU: Southern Africa Labour and Development Research Unit
SES: Socioeconomic status

## Footnotes

1 Plausible readings were considered as 50 mmHg ≤ SBP ≤ 250 mmHg, 30 mmHg ≤ DBP ≤ 150 mmHg, and mean SBP > mean DBP [48, 84].

2 Waist circumference measurements greater than 190.5 cm (75 in.) or less than 50.8 cm (20 in.) [85] were considered implausible.

3 Household incomes of zero were given an income of one so that the log would return a value of 0.
