# Supplementary material for: Income inequality and cardiovascular disease risk factors in a highly unequal country: a fixed-effects analysis from South Africa
Source: Int J Equity Health. 2018 Mar 6;17:31. doi: 10.1186/s12939-018-0741-0 (PMC5839065; doi:10.1186/s12939-018-0741-0)
Supplement: Supplementary file 1 — National and district council Gini coefficients. (DOCX 15 kb) [file 12939_2018_741_MOESM1_ESM.docx]

**Additional File 1. National and District Council Gini Coefficients**

| **District Council** | **Name (district municipality)** | **Gini, Community Survey 2007** | **Gini, Census 2011** |
| --- | --- | --- | --- |
|  | **South Africa** | **0.77** | **0.78** |
| **101** | West Coast | 0.66 | 0.70 |
| **102** | Cape Winelands | 0.70 | 0.74 |
| **103** | Overberg | 0.69 | 0.71 |
| **104** | Eden | 0.72 | 0.74 |
| **105** | Central Karoo | 0.69 | 0.73 |
| **199** | City of Cape Town | 0.76 | 0.74 |
| **210** | Cacadu | 0.76 | 0.76 |
| **212** | Amathole | 0.68 | 0.69 |
| **213** | Chris Hani | 0.69 | 0.75 |
| **214** | Ukhahlamba | 0.71 | 0.76 |
| **215** | O.R. Tambo | 0.74 | 0.77 |
| **244** | Alfred Nzo | 0.74 | 0.73 |
| **260** | Buffalo City | 0.78 | 0.78 |
| **299** | Nelson Mandela Bay Metro | 0.78 | 0.76 |
| **306** | Namakwa | 0.67 | 0.73 |
| **307** | Pixley ka Seme | 0.75 | 0.73 |
| **308** | Siyanda | 0.72 | 0.72 |
| **309** | Frances Baard | 0.76 | 0.74 |
| **345** | John Taolo Gaetsewe | 0.78 | 0.75 |
| **416** | Xhariep | 0.66 | 0.74 |
| **418** | Lejweleputswa | 0.66 | 0.73 |
| **419** | Thabo Mofutsanyane | 0.76 | 0.74 |
| **420** | Fezile Dabi | 0.71 | 0.75 |
| **499** | Mangaung | 0.72 | 0.76 |
| **521** | Ugu | 0.77 | 0.76 |
| **522** | UMgungundlovu | 0.76 | 0.76 |
| **523** | Uthukela | 0.71 | 0.78 |
| **527** | Umkhanyakude | 0.80 | 0.75 |
| **528** | Uthungulu | 0.72 | 0.76 |
| **543** | Sisonke | 0.76 | 0.77 |
| **554** | Umzinyathi | 0.65 | 0.72 |
| **555** | Amajuba | 0.70 | 0.78 |
| **556** | Zululand | 0.77 | 0.75 |
| **559** | iLembe | 0.70 | 0.75 |
| **599** | Ethekwini Municipality | 0.75 | 0.76 |
| **637** | Bojanala | 0.65 | 0.71 |
| **638** | Ngaka Modiri Molema | 0.74 | 0.77 |
| **639** | Dr. Ruth Segomotsi Mompati | 0.71 | 0.76 |
| **640** | Dr. Kenneth Kaunda | 0.71 | 0.77 |
| **742** | Sedibeng | 0.78 | 0.75 |
| **748** | West Rand | 0.71 | 0.72 |
| **797** | Ekurhuleni Metropolitan Municipality | 0.76 | 0.75 |
| **798** | City of Johannesburg Metropolitan Municipality | 0.79 | 0.77 |
| **799** | City of Tshwane | 0.78 | 0.75 |
| **830** | Gert Sibande | 0.74 | 0.77 |
| **831** | Nkangala | 0.76 | 0.74 |
| **832** | Ehlanzeni | 0.73 | 0.76 |
| **933** | Mopani | 0.70 | 0.77 |
| **934** | Vhembe | 0.69 | 0.74 |
| **935** | Capricorn | 0.73 | 0.78 |
| **936** | Waterberg | 0.72 | 0.74 |
| **947** | Greater Sekhukhune | 0.71 | 0.74 |

Gini coefficients based on equivalized household income using proxy midpoints of household derived income categories from South Africa’s Community Survey 2007 and Census 2011.
