# Supplementary material for: Income inequality and cardiovascular disease risk factors in a highly unequal country: a fixed-effects analysis from South Africa
Source: Int J Equity Health. 2018 Mar 6;17:31. doi: 10.1186/s12939-018-0741-0 (PMC5839065; doi:10.1186/s12939-018-0741-0)
Supplement: Supplementary file 2 — Additional details on variables used in analysis. (DOCX 22 kb) [file 12939_2018_741_MOESM2_ESM.docx]

**Additional File 2**. **Additional Details on Variables Used in Analysis**

Gini coefficient calculations:

Gini coefficients were calculated from household incomes in the Community Survey 2007 (CS 2007) and Census 2011. We derived household incomes in the CS 2007 from individual gross income. To be consistent with the procedure used by Statistics South Africa to derive household income in the Census 2011 [1], we allocated the Census 2011 “proxy midpoint” values to each income category for all individuals in the CS 2007 (see Table 3.1 below). We summed these midpoint values across all individuals in each household, and then re-categorized the household sum according to the original census and CS 2007 income categories. For the CS 2007 and Census 2011, we assigned each household an annual income for their derived household income categories based on the census proxy midpoint values and divided by 12 to approximate monthly household income. We equivalized household incomes by dividing by the square root of household size, following the OECD method [2], and deflated household incomes using August 2012—the NIDS Wave 3 modal month—as the base month, according to the Consumer Price Index (CPI) [3]. For the CS 2007, the deflator used was the average of the CPI headline indices for the months February 2006 through January 2007, corresponding to the 12 months preceding the CS 2007, which was conducted in February-March 2007 and asked income in reference to the preceding 12 months [4]. The deflator we used for incomes in the Census 2011 was the average CPI headline for the months of October 2010 through September 2011 which were the 12 months preceding census day (October 9^th^-10^th^, 2011). We calculated district-level Gini coefficients from the deflated monthly household income values using 2011 district boundaries and applying household weights.

Alternative district Gini coefficients calculated using total or per capita household income rather than equivalized household income, and using the numerical midpoint of the household income categories rather than the census proxy midpoints were highly correlated with those based on equivalized household income from census proxy midpoint values used in the main analysis [correlation coefficients >0.90 (p<0.0001) for Community Survey 2007 and >0.80 (p<0.0001) for the Census 2011].

**Table 3.1. Census Income categories and proxy values used to derive household income** (Stats SA 2014).

| **Census Income range/category (Rand)** | **Proxy value allocated (Rand)** |
| --- | --- |
| No income | 0 |
| 1-4,800 | 3,200 |
| 4,801-9,600 | 7,200 |
| 9,601-19,200 | 13,576 |
| 19,201-38,400 | 27,153 |
| 38,401-76,800 | 54,306 |
| 76,801-153,600 | 108,612 |
| 153,601-307,200 | 217,223 |
| 307,201-614,400 | 434,446 |
| 614,401-1,228,800 | 868,893 |
| 1,228,801-2,457,600 | 1,727,786 |
| 2,457,601 or more | 4,915,200 |

*Outcomes:*

Alcohol Consumption: We estimated alcohol consumption based on two questions in the adult survey. Respondents were was asked how often they consume alcohol (never, no longer, less than once a week, on 1 or 2 days a week, on 5 or 6 days a week, or every day). We coded those reporting drinking everyday as drinking 7 days a week. And those reporting never or no longer drinking as drinking 0 days a week. For the other categories, we took the midpoint. The number of standard drinks consumed per day of drinking was also asked and reported in the following categories: 13 or more standard drinks, 9 to 12 standard drinks, 7 to 8 standard drinks, 5 to 6 standard drinks, 3 or 4 standard drinks, or 1 or 2 standard drinks. For the categories that provided a range of standard drinks, we took the midpoint. For those reporting 13 or more standard drinks, we set the number of drinks to 13. We multiplied these estimates for days per week of drinking and number of drinks consumed per day of drinking to create a number of drinks consumed per week, and divided by 7 to estimate the number of drinks per day. Women who averaged more than 1 drink per day and men who averaged more than 2 drinks per day were classified as having high alcohol consumption. Data on alcoholic drinks per drinking occasion were not available to capture binge drinking as defined by the Centers for Disease Control and Prevention (CDC)[6]. However, we also classified respondents who reported drinking more than 4 standard drinks on a day when they drink alcohol (e.g., 5+ drinks) as having high alcohol consumption. In addition, women averaging 8 or more drinks per week and men averaging 15 or more drinks per week [heavy drinking as defined by the CDC [6]] were classified as having high alcohol consumption.

**References**

1. Stats SA. Census 2011 10% sample metadata. Pretoria: Stats SA; 2014.

2. OECD. What Are Equivalence Scales? www.oecd.org/social/inequality.htm (N.d.). Accessed 2 Aug 2015.

3. Stats SA. CPI history: 1960 onwards. 2015. http://www.statssa.gov.za/publications/P0141/CPIHistory.pdf? Accessed 2 Aug 2015.

4. Stats SA. Community Survey, 2007 (Revised version). Pretoria: Stats SA; 2007.

5. De Villiers L, Brown M, Woolard I, Daniels R, Leibbrandt M. National Income Dynamics Study wave 3 user manual. Cape Town: Southern Africa Labour and Development Research Unit; 2014.

6. CDC. Fact Sheets - Alcohol Use and Your Health. http://www.cdc.gov/alcohol/fact-sheets/alcohol-use.htm (2014). Accessed 15 Jan 2015.
