# Supplementary material for: Income inequality and cardiovascular disease risk factors in a highly unequal country: a fixed-effects analysis from South Africa
Source: Int J Equity Health. 2018 Mar 6;17:31. doi: 10.1186/s12939-018-0741-0 (PMC5839065; doi:10.1186/s12939-018-0741-0)
Supplement: Supplementary file 4 — Baseline characteristics for individuals excluded from the sample. (DOCX 20 kb) [file 12939_2018_741_MOESM4_ESM.docx]

**Additional File 4. Baseline characteristics for individuals excluded from the sample**

|  | **N** | **Proportion/**  **Mean (standard deviation)** |
| --- | --- | --- |
| Total | 7,515 |  |
| Female | 3,852 | 52.0% |
| Race/population group  *African*  *Coloured*  *Asian/Indian*  *White* | 5,515  1,088  151  651 | 74.5%  14.7%  2.0%  8.8% |
| Age (years) | 7,405 | 35.8 (17.9) |
| Highest education level  *No education*  *Some general education & training*  *General education & training*  *Some further education & training*  *Further education & training*  *Higher education* | 838  2,113  666  1,762  1,398  554 | 11.4%  28.8%  9.1%  24.0%  19.1%  7.6% |
| Employment status  *Employed*  *Unemployed*  *Not economically active* | 2,338  1,102  2,687 | 38.2%  18.0%  43.9% |
| Marital Status  *Currently married/cohabiting*  *Currently single* | 2,102  4,153 | 33.6%  66.5% |
| Mean household size^a^ | 1,971 | 2.7 (1.9) |
| Household receipt of government grants^a^ | 699 | 35.7% |
| Monthly household income (Rand) ^a^ | 1,971 | 7,007.7 (12,507.0) |
| Rural household^a^ | 767 | 38.9% |
| Systolic blood pressure (mm Hg) | 5,460 | 123.7 (22.4) |
| Diastolic blood pressure (mm Hg) | 5,460 | 79.4 (13.7) |
| Body mass index (kg/m^2^) | 5,410 | 25.0 (6.6) |
| Waist circumference (cm) | 5,360 | 84.3 (15.3) |
| Current smoker | 1,460 | 23.4% |
| Physical inactivity | 4,202 | 67.6% |
| High alcohol consumption | 794 | 12.8% |

1. Descriptive characteristics listed here are based on the number of households and not the number of individuals
